# Tobacco impact on quality of life, a cross-sectional study of smokers, snuff-users and non-users of tobacco

**DOI:** 10.1186/s12889-023-15844-z

**Published:** 2023-05-15

**Authors:** Solbrith Wachsmann, Lena Nordeman, Annika Billhult, Gun Rembeck

**Affiliations:** 1 Närhälsan Primary Health Care Ängabo, Alingsås, Region Västra Götaland Sweden; 2 Research, Education, Development and Innovation, Center Södra Älvsborg, Borås, Region Västra Götaland Sweden; 3 Department Rehabilitation and Health, Institute of Neuroscience and Physiology, University of Gothenburg, Gothenburg, Sweden; 4 Department of Health Sciences Faculty of Caring Science, Work Life and Social Welfare, University of Borås, Borås, Sweden; 5 Borås Youth Guidance Centre, Regional Health, Borås, Region Västra Götaland Sweden; 6 Primary Health Care, School of Public Health and Community Medicine, Institute of Medicine, Sahlgrenska Academy, University of Gothenburg, Gothenburg, Sweden

**Keywords:** Health, Population characteristics, Quality of life, Smokeless tobacco

## Abstract

**Background:**

Tobacco smoking is a major public health issue, and also affects health-related quality of life. There has been considerable debate as to whether oral moist snuff, a form of tobacco placed in the oral cavity between the upper lip and gum as in sublabial administration, can be considered a safe alternative to smoking. The aim of this study was to investigate the association between health-related quality of life and smoking, snuff use, gender and age.

**Method:**

This cross-sectional study included 674 women and 605 men aged 18 to 65 recruited through a Swedish population database. Subjects completed a questionnaire about tobacco use and the 36-item Short Form Health Survey (SF-36). Multivariable logistic regression analyses were performed for the association between health-related quality of life and tobacco use, gender and age. The median perceived health-related quality of life (SF-36) for an age-matched Swedish population was used as the cutoff: above the cutoff indicated better-than-average health coded as 1, or otherwise coded as 0. The independent variables were smoking (pack-decades), snuff-use (box-decades), gender and age in decades. The outcome was presented as the Odds Ratio (OR) with a 95% confidence interval (CI) for each independent variable.

**Results:**

The experience of cigarette smoking is associated with decreased physical functioning (PF), general health (GH), vitality (VT), social functioning (SF) and mental health (MH) as well as both lower physical component summary (PCS) and mental component summary (MCS). Further, the experience of snuff use is associated with bodily pain (BP), lower VT, and lower PCS. In the study population older age is associated with lower PF,GH, VT, MH, PCS and MCS. Female gender is associated with lower PF and VT.

**Conclusion:**

This study shows that smoking is associated with lower health-related quality of life. The results also illuminate the detrimental health effects of using snuff, implying that snuff too is a health hazard. As studies on the bodily effects of snuff are relatively scarce, it is imperative that we continue to address and investigate the impact on the population using snuff on a regular basis.

**Trial registration:**

ClinicalTrials.gov ID NCT05409963 05251022 08/06/22.

## Background

Tobacco smoking is the leading preventable risk factor for disease and suffering in the world. In Sweden, about 12 000 people die due to tobacco smoking, and hundreds of thousands become ill annually from cardiovascular disease, chronic obstructive pulmonary disease (COPD) and cancer [1]. Tobacco use has been declining in many countries for several years. In 2020, 7% of the population in Sweden were active smokers, and 5% of women and 19% of men used snuff daily [2]. However, there are also many countries where the proportion of smokers is much higher. The financial burden of tobacco use is extensive world-wide, 1436 billion US-dollar equivalent to 1.8% of the world's annual gross domestic product (GDP) [3]. In Sweden the financial burden is 75 billion SEK per year [4].

### Use of snuff and its relationship to health

There has been considerable debate as to whether oral moist snuff, a form of tobacco that is placed in the oral cavity rather than being smoked, can be seen as a safe alternative to smoking [5]. Snuff is often portrayed as relatively harmless, and tobacco companies present snuff to smokers as a less dangerous alternative to smoking. Snuff contains 2000 different ingredients, and "wet-snuff" (unlike cured tobacco) is banned from being sold in the European Union (EU) except in Sweden [6]. Studies on the effects of snuff on health have shown that mortality among men in Sweden using snuff is 28% higher than for men who do not use snuff [7]. High consumption of snuff predicts an increased risk of type 2 diabetes [8], risk of heart failure [9], a higher risk of oral cancer [10], and an increased risk of stillbirth by 60% [11]. Snuff users in Sweden had poorer health than those who did not use tobacco, but their health was less impaired than smokers [5].

### Health-related quality of life

Health-related quality of life, based on an individual’s perspective, is often measured to gain understanding of how people’s lives are affected by various illnesses and what benefits and limitations medical care may have. Health-related quality of life indicates the subjective value of satisfaction in life that people are experiencing. This is affected by individual needs, expectations, physical and mental functioning, the person’s relationships to others, and to social and material standards [12]. The World Health Organization (WHO) defines quality of life as a subjective assessment of one’s life situation on three levels: physical, mental and social. Perception is influenced by a number of factors such as the physical environment, occupational satisfaction, education, social and intellectual satisfaction, freedom, justice, and freedom from oppression [13]. Low health-related quality of life can lead to poorer health [14] and vice versa. However, poorer health does not need to result in low health-related quality of life [15].

### The remaining dilemma

Research has shown that smokers have a poorer health-related quality of life than non-smokers [16], and that women smokers have a poorer quality of life than male smokers [17]. Snuff is now portrayed as a harmless or less dangerous alternative to smoking. We know that snuff is associated with some impairments in health but there are no studies investigating the association between snuff and health-related quality of life. The aim of this study was to investigate the association between health-related quality of life and tobacco use (smoking or use of snuff), gender and age.

## Methods

This was a cross-sectional study using a questionnaire for investigating tobacco use and the questionnaire 36-item Short Form Health Survey (SF-36) version 1.

Ethical approval was granted by the Regional Ethics Review Board in Gothenburg, Dnr: 367–10 and the study was registered in ClinicalTrials.gov ID NCT05409963 05251022 08/06/22.

### Study population

Three thousand women and men, 18–65 years old, were randomly selected from the Swedish population database (SPAR) taken from a mixed urban and rural area in the southwestern part of Sweden.

### Data collection

Participants were mailed an invitation to enroll in the study, a consent form and two questionnaires, one about tobacco use and the SF-36 version 1, along with a return envelope. Two reminders were sent.

Demographic data such as age and gender were collected. The questionnaire about tobacco use consisted of items with fixed response alternatives categorized according to: never used tobacco, previously smoked, currently smokes not using snuff, previously used snuff, currently uses snuff not smoking, previously smoked and used snuff, previously smoked and currently uses snuff, currently smokes and previously used snuff and currently smokes and uses snuff. Also, the number of years of use and cigarettes per day or snuff boxes per week, when applicable, was included. Face validity of the tobacco use questionnaire was determined by a pilot administration to eight subjects. These eight subjects found the questions easy to understand and no subsequent changes were made.

The SF-36 is a validated questionnaire grounded in WHO’s health definition of quality of life. It is generalizable to different contexts and has been used in over 4000 different studies and translated into Swedish in 1995 [18, 19]. The SF-36 collected data on perceived health status in eight domains: physical functioning (PF), role-physical (RP), bodily pain (BP), general health (GH), vitality (VT), social functioning (SF), role-emotional (RE) and mental health (MH). The scores in the eight domains can be combined to calculate more comprehensive indicators for physical and mental health: the physical component summary (PCS) and mental component summary (MCS). Scores range from 0 to 100 with higher scores representing a higher perceived health [18].

### Statistical analysis

The term pack-year (numbers of cigarettes / day * number of years / 20) and box-year (numbers of snuff boxes / week * number of years) was used to describe tobacco use. The variables smoking and snuff use were transformed from one pack-year to pack-decades by dividing pack-year by 10. Similarly, box-years were transformed to box-decades and age from years to decades.

Multivariable logistic regression analyses were performed for the association between quality of life and tobacco use, gender and age [20]. The dependent variable was each of the eight domains in SF-36 (PF, RP, BP, GH, VT, SF, RE, MH), and the summary component scores PCS and MCS. The median for an age-matched Swedish population was used as the cutoff [21], above the cutoff indicated better-than-average health coded as 1, or otherwise coded as 0. The independent variables were smoking (pack-decades), snuff-use (box-decades), gender and age in decades. Odds Ratio (OR) with a 95% confidence interval (CI) was calculated for each independent variable.

To validate each model, Nagelkerke R square, Area under the Receiver Operating Characteristic curve (AUC), the Omnibus test of model (X^2^ *p* < 0.05) and the Hosmer and Lemeshow test (*p* > 0.05) were performed and evaluated. The Statistical Package for Social Science (SPSS) for Windows, version 25 was used for statistical analyses. The level of significance was set at 0.05.

## Results

An invitation was sent out to 3,000 individuals and 1,279 (43%) responded, 674 women and 605 men aged 18 to 66 years. Their mean age was 44 (SD 14). Forty-five percent had at some time used tobacco (Table 1).

**Table 1:** Tobacco use among participants

	Men (*n* = 605)		Women (*n* = 674)		All (*n* = 1279)	
	n	%	n	%	n	%
Never used tobacco	278	46	426	63	704	55
Previously smoked	83	14	147	22	230	18
Currently smokes not using snuff	47	7.8	70	10	117	9.1
Previously used snuff	44	7.3	4	0.59	48	3.8
Currently uses snuff not smoking	46	7.6	6	0.89	52	4.1
Previously smoked and used snuff	37	6.1	1	0.15	38	3.0
Previously smoked and currently uses snuff	45	7.4	11	1.6	56	4.4
Currently smokes and previously used snuff	14	2.3	5	0.74	19	1.5
Currently smokes and uses of snuff	11	1.8	4	0.60	15	1.2

All participants rated their health-related quality of life as comparable to the average Swedish population (Table 2).

**Table 2:** Health related quality of life of participants compared to the Swedish population

	**Male participants (*n* = 597)**	**Swedish male population (*n* = 4268) **[1]	**Female participants (*n* = 669)**	**Swedish female population (*n* = 4592) **[1]	**Male and female participants (*n* = 1266)**	**Swedish male and female (*n* = 8930) **[1]
**Dimension of, Short form Health Survey(SF-36) (range 0–100)**	**Median (Q1-Q3)**	**Median (Q1-Q3) < **	**Median (Q1-Q3)**	**Median (Q1-Q3)**	**Median (Q1-Q3)**	**Median (Q1-Q3)**
Physical Function	95 (90–100)	100 (90–100)	95 (85–100)	95 (80–100)	95 (90–100)	95 (85–100)
Role Physical	100 (100–100)	100 (75–100)	100 (75–100)	100 (75–100)	100 (100–100)	100 (75–100)
Bodily Pain	84 (61–100)	84 (61–100)	84 (52–100)	80 (51–100)	84 (61–100)	84 (52–100)
General Health	82 (67–92)	82 (67–94)	82 (62–92)	82 (62–92)	82 (62–92)	82 (62–92)
Vitality	70 (55–85)	75 (60–85)	65 (45–80)	70 (50–85)	70 (50–80)	75 (55–85)
Social Functioning	100 (88–100)	100 (88–100)	100 (75–100)	100 (75–100)	100 (84–100)	100 (87–100)
Role Emotional	100 (100–100)	100 (100–100)	100 (100–100)	100 (67–100)	100 (100–100)	100 (100–100)
Mental Health	84 (76–92)	88 (76–96)	84 (72–92)	84 (68–92)	84 (72–92)	88 (72–96)
Physical component summary	53 (48–56)	54 (48–57)	53 (47–56)	53 (45–56)	53 (48–56)	53 (46–57)
Mental component summary	53 (47–56)	57 (48–57)	52 (44–55)	53 (45–57)	52 (45–56)	53 (46–57)

The experience of cigarette smoking is associated with decreased physical functioning, general health, vitality, social functioning and mental health as well as both lower physical component summary and mental component summary. Further, the experience of snuff use is associated with bodily pain, lower vitality, and lower physical component summary. Use of snuff was also in some aspects associated with a lower quality of life, but to a lesser extent than for smokers. In the study population older age is associated with lower PF,GH, VT, MH, PCS and MCS. Female gender is associated with lower PF and VT (Table 3).

**Table 3:** Association between quality of life and tobacco use adjusted for age and gender

	Physical Functioning ≥ 88^a^		Bodily Pain ≥ 75^a^		General Health ≥ 76^a^		Vitality ≥ 69^a^	
	(*n* = 1246)		(*n* = 1244)		*(n* = 1247)		(*n* = 1244)	
	OR (95%CI)	*p*-value	OR (95%CI)	*p*-value	OR (95%CI)	*p*-value	OR (95%CI)	*p*-value
Pack-year 10	0.72 (0.64–0.82)	5.4 × 10 ^−7^	0.89 (0.79–1.0)	0.056	0.75 (0.66–0.85)	0.000005	0.71 (0.62–0.81)	1.3 × 10 ^−7^
Box-year 10	0.99 (0.96–1.0)	0.53	0.95 (0.91–0.98)	0.00078	0.99 (0.96–1.0)	0.29	0.96 (0.93–0.99)	0.0066
Female gender	0.58 (0.43–0.79)	0.00042	0.77 (0.61–0.98)	0.033	0.85 (0.67–1.1)	0.20	0.64 (0.50–0.81)	0.00027
Age 10 yr	0.70 (0.62–0.78)	3.3 × 10 ^−10^	0.92 (0.85–1.0)	0.058	0.91 (0.83–0.99)	0.03	1.1 (1.1–1.2)	0.0017
Model validation								
**Overall performance**								
Nagelkerke R Square	0.12		0.027		0.041		0.048	
**Discrimination**								
AUC^b^	0.69 (0.65–0.72)	6.9 × 10 ^−22^	0.58 (0.55–0.61)	8.7 × 10 ^−7^	0.60 (0.56–0.63)	1.1 × 10 ^−8^	0.60 (0.57–0.63)	2.4 × 10 ^−10^
**Calibration**^**c**^								
Omnibus test	102 (4)	4.1 × 10 ^−21^	26 (4)	0.000034	38 (4)	1.3 × 10 ^−7^	46 (4)	2.7 × 10 ^−9^
Hosmer and Lemeshow test	2.0 (8)	0.98	7.1 (8)	0.53	7.8 (8)	0.46	11 (8)	0.18
	Social Function ≥ 89^a^		Mental Health ≥ 81^a^		Physical component summary		Mental component summary	
	(*n* = 1244)		(*n* = 1244)		≥ 50^a^ *(n* = 1202)		≥ 50^a^ (*n* = 1185)	
	OR (95%CI)	*p*-value	OR (95%CI)	*p*-value	OR (95%CI)	*p*-value	OR (95%CI)	*p*-value
Pack-year 10	0.82 (0.72–0.92)	0.0011	0.77 (0.68–0.87)	0.000038	0.84 (0.74–0.95)	0.0049	0.77 (0.68–0.87)	0.000051
Box-year 10	0.98 (0.96–1.0)	0.24	0.98 (0.96–1.0)	0.25	0.96 (0.93–0.99)	0.0086	1.00 (0.97–1.0)	0.79
Female gender	0.84 (0.66–1.1)	0.17	0.87 (0.68–1.1)	0.24	0.87 (0.67–1.1)	0.31	0.80 (0.62–1.0)	0.073
Age 10 yr	1.0 (0.96–1.1)	0.31	1.2 (1.1–1.3)	0.000002	0.77 (0.70–0.85)	2.4 × 10 ^−7^	1.3 (1.2–1.4)	1.5 × 10 ^−7^
Model validation								
**Overall performance**								
Nagelkerke R Square	0.014		0.034		0.067		0.043	
**Discrimination**								
AUC^b^	0.55 (0.52–0.59)	0.0018	0.60 (0.56–0.63)	6.3 × 10 ^−9^	0.63 (0.60–0.67)	3.6 × 10 ^−13^	0.61 (0.57–0.64)	1.1 × 10^–9^
**Calibration**^**c**^								
Omnibus test	13 (4)	0.014	32 (4)	0.000002	58 (4)	8.0 × 10 ^−12^	37 (4)	1.4 × 10^–7^
Hosmer and Lemeshow test	6.6 (8)	0.58	7.8 (8)	0.45	5.7 (8)	0.69	3.4 (8)	0.91

^a^The median for an age matched Swedish population is used as cut off, above cut off indicates better than average health

^b^Area under curve (95% confidence interval) *P*-value to the right of the confidence interval

^c^Chi square (degree of freedom) *P*-value to the right of df

## Discussion

As expected, it was found that smoking was associated with reduced perceived health. A new finding is that the use of snuff is associated with a lower quality of life in the domains of Bodily Pain and Vitality (SF-36).

### Strengths and weaknesses

A strength of the study is that initial recruitment was carried out via random selection from the population register. The response rates in previous studies sending questionnaires to participants have been reported at 21—49 percent [22]. The response rate in this study was 42.6 percent, which is at the higher end of what could be expected.

The ability for cross-sectional studies to establish the cause and effect relationship is limited. However, in this particular case, it is the best option available since randomized controlled trials cannot be carried out. This study did not register profession, educational level or work status. It is possible that any of these variables might be associated with a lower quality of life. However, the fact that the sample in this study had a quality of life equal to the average Swedish population suggests there was no selection bias in respect to profession, educational level or work status.

Nagelkerke R square and area under curve for the different models are generally low, indicating that perceived quality of life is, to a substantial extent, explained by other variables than those included in our models. However, the models are better than pure chance, and as such, valid for comparing the relative importance of tobacco use, gender and age on perceived quality of life.

### Use of snuff and its association to quality of life

Musculoskeletal pain is more prevalent in patients who smoke [23]. Moreover, smoking has been considered a risk indicator for sciatica and low back pain. Possible explanations are that nicotine reduces blood flow to the spinal discs [24] which leads to pain. Snuff contains 20 times more nicotine compared to cigarettes [25]. This effect on bodily pain was seen in this study in snuff users. The results of the present study show similar results as an older study on the detrimental health effects of using snuff [5], implying that this is a continuous health hazard.

### Gender association to health-related quality of life

The women in this study reported significantly lower scores in the domains BP and VT, and especially in PF. Our findings are consistent with previous reports stating that men report health-related quality of life higher than women [18]. There is a multitude of possible explanations for this gender inequality.

Menstruation and ovulation may cause pain in the ovaries and uterus to varying degrees, but the menstrual cycle should not affect aspects of quality of life other than bodily pain, measured by the domain BP in SF-36 [26]. Female diseases such as endometriosis, which occur in about 200,000 to 250,000 in Sweden [27] affecting mainly younger women, results in lower health-related quality of life in all eight domains of SF-36, but mostly for the domain BP [26]. Also, women’s menopause may explain some gender differences in bodily pain, sleeping disturbance, which affects the physical function of vitality [28]. However, many increases in ailments among women aged 45–55, such as pain, sleep disorders, physical and mental fatigue, do not have to be related to menopause [29]. Being informal caregivers such as caring for elderly relatives, often leads to lower physical health and it is mostly women who are the informal caregivers [30]. We also know that women often have poorer economic conditions than men, even if they have a higher level of education [2].

Twenty-six percent of women described being victims of some kind of sexual violence as a child as opposed to 11% of men. Eighteen percent of Swedish women have described being currently subjected to violence from partners or former partners. Twenty-two percent of Swedish women state that they have been subjected to sexual violence as opposed to 4.5% of men [31]. It is not difficult to imagine that these experiences may lead to decreased health-related quality of life in the SF-36 domains VT and PF.

## Conclusion

This study shows that the use of tobacco, both smoking cigarettes and using snuff, is associated with lower health-related quality of life. The results also show that using snuff is associated with a lower quality of life in the domains of Bodily Pain and Vitality (SF-36), implying that it is a continuous health hazard. As studies on the bodily effects of snuff are relatively scarce, it is imperative that we continue to address and investigate the impact on the population using snuff on a regular basis.

## Data Availability

The datasets used and/or analyzed during the current study are available from the corresponding author upon request.
